# Current State of Stroke Care in the Philippines

**DOI:** 10.3389/fneur.2021.665086

**Published:** 2021-08-17

**Authors:** ME. V. Collantes, Y. H. Zuñiga, C. N. Granada, D. R. Uezono, L. C. De Castillo, C. G. Enriquez, K. D. Ignacio, S. D. Ignacio, R. D. Jamora

**Affiliations:** ^1^Department of Neurosciences, College of Medicine, Philippine General Hospital, University of the Philippines, Manila, Philippines; ^2^University of the Philippines, Manila, Philippines; ^3^Department of Health, Manila, Philippines; ^4^College of Public Health, University of the Philippines, Manila, Philippines; ^5^Department of Rehabilitation Medicine, College of Medicine, Philippine General Hospital, University of the Philippines, Manila, Philippines

**Keywords:** stroke, health systems, policy, prevention and management, health system

## Abstract

Stroke remains the leading cause of disability and death in the Philippines. Evaluating the current state of stroke care, the needed resources, and the gaps in health policies and programs is crucial to decrease stroke-related mortality and morbidity effectively. This paper aims to characterize the Philippines' stroke system of care and network using the World Health Organization health system building blocks framework. To integrate existing national laws and policies governing stroke and its risk factors dispersed across many general policies, the Philippine Department of Health (DOH) institutionalized a national policy framework for preventing and managing stroke. Despite policy reforms, government financing coverage remains limited. In terms of access to medicines, the government launched its stroke medicine access program (MAP) in 2016, providing more than 1,000 vials of recombinant tissue plasminogen activator (rTPA) or alteplase subsidized to selected government hospitals across the country. However, DOH discontinued the program due to the lack of neuroimaging machines and organized system of care to support the provision of the said medicine. Despite limited resources, stroke diagnostics and treatment facilities are more concentrated in urban settings, mostly in private hospitals, where out-of-pocket expenditures prevail. These barriers to access are also reflective of the current state of human resource on stroke where medical specialists (e.g., neurologists) serve in the few tertiary and training hospitals situated in urban settings. Meanwhile, there is no established unified national stroke registry thus, determining the local burden of stroke remains a challenge. The lack of centralization and fragmentation of the stroke cases reporting system leads to reliance on data from hospital records or community-based stroke surveys, which may inaccurately depict the country's actual stroke incidence and prevalence. Based on these gaps, specific recommendations geared toward systems approach - governance, financing, information system, human resources for health, and medicines were identified.

## Introduction

The Philippines is an archipelagic nation with over 7,100 islands divided into three major island groups - Luzon, Visayas, and Mindanao, with its capital Manila located on the largest island Luzon (1). With over 109 million Filipinos living in the country, its population is generally young, with almost 40% belonging to the age group below 19 years old and only 5% are aged 65 and above (2).

From 2009 to 2019, stroke remains the second leading cause of death and one of the top five leading causes of disability in the Philippines (3). The true stroke prevalence is uncertain, but reported estimates vary between 0.9% (2005) (4) to 2.6% (2017) of the population (5). Based on types of stroke, seven out of 10 cases are diagnosed as ischaemic while the other three are considered hemorrhagic (4). Thirty six percent (36%) of the total stroke deaths are not attended by any medical personnel (6).

The Philippines' Local Government Code of 1991 has resulted in the devolution of different health services in the country, transferring the management of health systems from the national level to the provincial, city, and municipal level or the local government units (LGUs) (7). Thus, health outcomes varied from one LGU to another. Coping to the new responsibilities that came with devolution posed a challenge to some LGUs, leaving some health facilities poorly equipped and staffed, thereby affecting the quality of health services.

Contributing further to this challenge is the country's archipelagic nature, making health services delivery even more difficult. Geographically isolated and disadvantaged areas have limited access to health facilities. Added to this burden is the migration of health professionals to other countries searching for better wages, compromising the health care delivery (8). These health system challenges can compromise stroke care of the country, negatively affecting the outcomes of the patients and are reflected in the national data.

In resource-limited settings like the Philippines, reporting comprehensive documentation of the current state of stroke care and identifying existing gaps and challenges can support the prioritization of measures to reduce the country's stroke-related mortality and morbidity. This paper aims to characterize the stroke care system in the Philippines using the World Health Organization (WHO) building blocks of the health system framework (9).

## Leadership and Governance

The Philippine Department of Health (DOH) has recently enacted Administrative Order No. 2020-0059 or the National Policy Framework on the Prevention, Control and Management of Acute Stroke in the Philippines. The policy aims to develop protocols for diagnosis, treatment, related care, support, and establishment of referral pathways that are cost-effective and widely used. The policy further seeks to build capacity for acute stroke management and establish Acute Stroke Ready Hospitals (10). This aligns with the preceding policy DOH Department Order 2017-0290 which provides for establishing brain centers and acute stroke units in selected DOH hospitals (11). Likewise, DOH will strengthen its health promotion and communication alongside a National Stroke Registry integrated into the Unified Disease Registry System (10).

National policies and legislations are also in place to address the risk factors associated with non-communicable diseases, including stroke. For instance, DOH developed a national multisectoral plan and a strategic action plan for NCD prevention for the years 2017–2025. Alongside this is the Philippine Plan of Action for Nutrition for 2017–2022, which includes the issues of overweight and obesity. The Philippine government also implemented tobacco and alcohol taxation through two republic acts in 2012 and 2017 (12).

Other policies related to stroke and its risk factors were also identified to be dispersed throughout many general policies such as in the Department of Health Administrative Order No. 2011-0003 or the National Policy on Strengthening the Prevention and Control of Chronic Lifestyle Related Non-Communicable Diseases (13), and the Administrative Order No. 2015-0052 or the National Policy on Palliative and Hospice Care in the Philippines (14).

## Financing

Navarro et al. estimated that about half a million Filipinos would be affected by stroke, costing about $350 million−1.2 billion in medical care. Financing health services for stroke remains a deterrent to timely treatment and management as costs are mainly out-of-pocket in terms of its curative and rehabilitative aspect coupled with the highly privatized nature of health care (4).

The Philippine Health Insurance Corporation (PhilHealth), the country's national health insurance system, reimburses only USD 560 (Php 28,000) and USD 760 (Php 38,000) for ischemic and hemorrhagic stroke, respectively, both of which cover professional and healthcare institution fees (15). Thrombolysis, the standard of care in acute ischemic stroke, includes costs for emergency plain cranial CT scan, urgent laboratory exams, medication cost (e.g., recombinant tissue plasminogen activator or rTPA), emergency room, and physician fees. Based on a survey conducted by the Stroke Society of the Philippines, the cost of thrombolysis can reach as much as USD 2,733 to USD 4,573 (Php 136,688–Php 228,678) in private hospitals while the range of expenditures in government hospitals cost between USD 65 to USD 718 (Php 3,239–Php 35,903). In a study by Diestro, Omar, and Sarmiento (16), the median in-hospitalization cost of stroke in a tertiary public hospital in the Philippines was calculated at USD 343 (Php 17,141.50) while the 90th percentile can be as high as USD 1,839.57 (Php 95,693.5). The expenses incurred in the private hospitals alone can cost several months of salary based on the average annual Filipino family income of USD 6,260 (Php 313,000). These costs can even become catastrophic, particularly in families belonging to the lower-income classes (17).

On the other hand, rehabilitation costs after stroke can range from USD 53.50 to as much as USD 4,591.60, based on a 2015 study by Akhavan Hejazi et al. in Malaysia. The costs include those for attendant care, medical aid, travel expenses, medical fees, and out-of-pocket expenses (18). These costs can again push a patient into poverty, especially those in the lower-income class. In the Philippines, financial coverage of rehabilitation services is considered insufficient due to the absence of a dedicated benefit package for these types of services. Some private health insurance companies may cover rehabilitation services, yet the total allowed number of claims for reimbursement is limited (19). This limitation indicates accessibility issues on rehabilitation services, specifically to the lower-income groups, as financing these services is mainly borne out of pocket.

In terms of preventive services related to stroke, PhilHealth mentions the inclusion of regular blood pressure measurements, counseling for lifestyle modification and smoking cessation, and several drugs for hypertension management such as amlodipine and losartan in its primary care benefit packages. Nicotine replacement therapy has already been included in the Philippine National Formulary Manual for Primary Healthcare, but its benefit package under PhilHealth has yet to be developed (20). On the other hand, prevention interventions such as traditional and mass media campaigns and implementation of policies targeting behavioral risk factors are then lodged within the Department of Health's budget.

## Access to Essential Medicines

The Philippine National Formulary (PNF) guides healthcare practitioners on the rational use of medicines and information on which drugs they can reimburse to the country's national health insurance system. Based on the 8th Edition of PNF, several drugs indicated for the prevention and management of stroke have included warfarin, aspirin, rTPA, clopidogrel, and dipyridamole (21). However, access to these medicines in the form of subsidy or coverage is limited to government hospitals that can procure these PNF-listed drugs. In 2016, the government implemented its stroke medicine access program (MAP), providing more than 1,000 vials of rTPA to the prioritized indigent population through selected government hospitals designated as access sites. To date, DOH halted the utilization of these drugs due to a lack of capital outlay and equipment such as computed tomography (CT) scan and the manpower support in the use of these drugs (22).

## Service Delivery

In the Philippines, hospitals are classified based on ownership (i.e., government or private), the scope of services (specialty or general), and functional capacity (i.e., Level I, II, or III) (8). There are 1,224 hospitals nationwide, translating to a total hospital bed capacity of 1,016,888 beds (8, 22). Private hospitals account for 53% (54,317) of the country's total bed capacity while the government-owned hospitals comprise the remaining 47% (47,371) (8). With limited facilities, the country only has 53 acute stroke ready hospitals or Level II (departmentalized hospital) and Level III (teaching and training) hospitals that meet the three requirements: 24/7 access to CT scan, an acute stroke/brain attack team and access to rTPA. On the other hand, the 47 acute stroke units have the same characteristics as those of acute stroke ready hospitals with the addition of specialized units designated for acute stroke patients with continuous monitoring of vital parameters and a multidisciplinary team approach including specialist nursing staff, most of which are in the highly urbanized cities (8, 23).

Aside from acute stroke ready hospitals and units, only 452 rehabilitation centers cater to 148.1 stroke cases per 100,000 population (24, 25). One of the difficulties of stroke rehabilitation in the Philippines is the access and availability of adequate rehabilitation facilities. The majority of these stroke rehabilitation facilities are in Metro Manila and other regions in the Luzon island. With this unequal distribution, Filipinos living in geographically isolated and disadvantaged areas face such barriers to timely post-stroke care (24). To note, only 15.8% of the country's hospitals have rehabilitation units. In a nationwide survey of hospitalized stroke patients, only half of the patients (54.1%) get referred to rehabilitation, with the median number of days from admission to referral being 3 days. Aside from inadequate access and facilities, the reasons for discontinuing therapy included cost and physician's decision to defer therapy due uncontrolled blood pressure, cardiovascular instability and physical inability of patients to tolerate early active treatment. The same study found that clients with hemorrhagic strokes were significantly less likely to be referred to rehabilitation than patients with cerebral infarcts (19).

**Figure 1:**
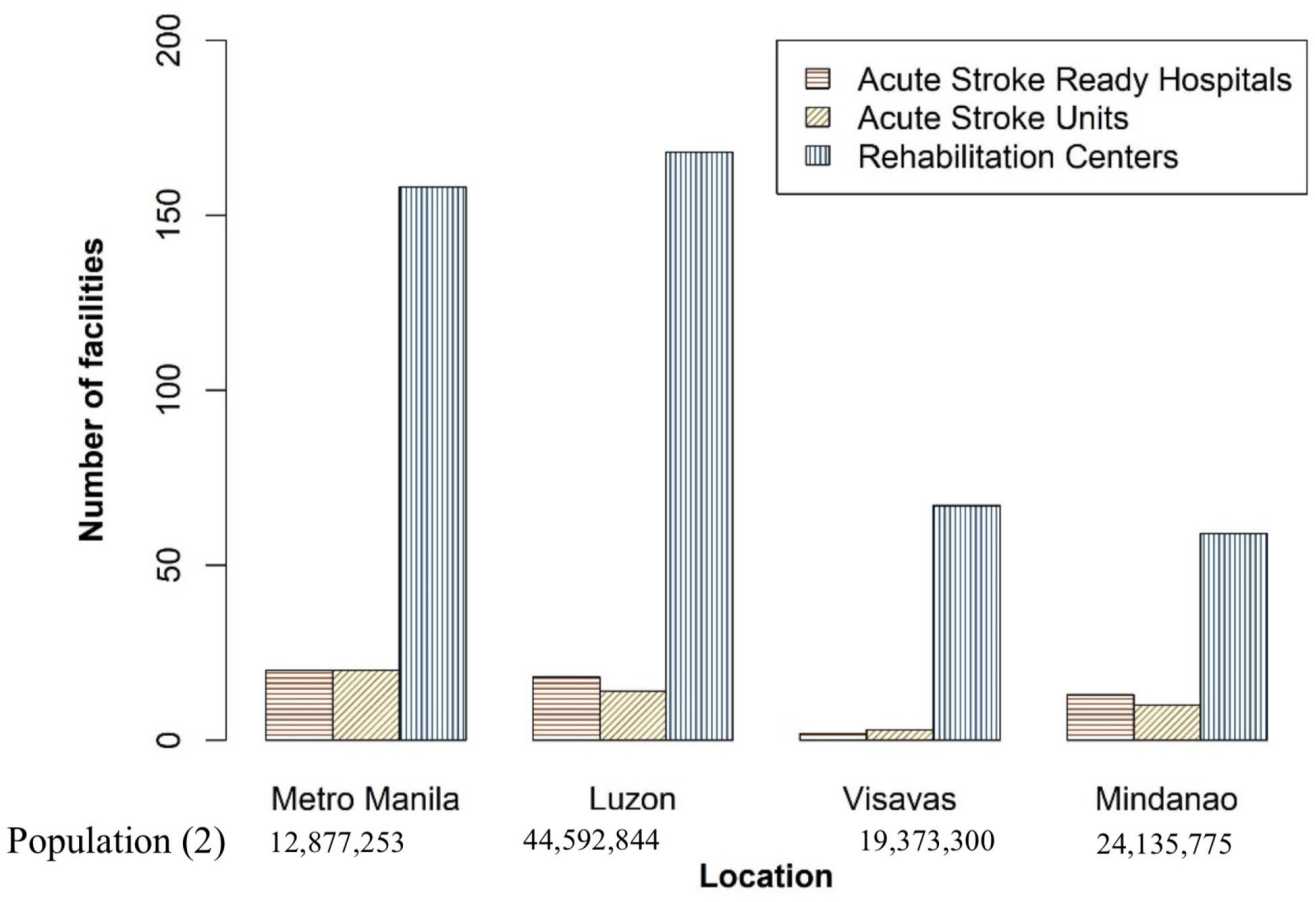
Distribution of stroke care centers in the Philippines, 2020 (24).

Figure 1 highlights the distribution of stroke facilities for treatment and rehabilitation across Metro Manila and the three island groups (24).

On the other hand, reported density of stroke diagnostic equipment such as computed tomography (CT) scan and magnetic resonance imaging (MRI) are low (1.09 per million population and 0.30 per million population, respectively) based on the 2013 WHO data on medical devices. Among Southeast Asian nations, Brunei, Thailand, and Singapore have relatively higher densities than the Philippines. The disparity grows bigger when compared to high-income countries such as Japan, Korea, and Canada (Table 1).

**Table 1:** The density of stroke diagnostic equipment (per million population) in ASEAN and other high-income countries (based on WHO Global Health Observatory as of 2013) (26).

**Country**	**CT per million population**	**MRI per million population**
Brunei	7.18	2.39
Cambodia	1.19	0.07
Indonesia	NR	NR
Lao PDR	0.74	0
Malaysia	6.43	2.89
Myanmar	0.08	0.08
Singapore	8.87	7.76
Thailand	5.95	NR
Vietnam	NR	NR
Philippine	1.09	0.30
Canada	13.76	7.99
Japan	101.20	45.94
South Korea	35.38	19.99

*CT, computed tomography; MRI, magnetic resonance imaging; NR, not reported*.

Emergency medical services (EMS) play a vital role in the management of stroke cases. However, EMS in the country is perceived to be fragmented and unstandardized (27). In an effort of the Philippine government to institutionalize emergency hotline 911 as the nationwide emergency answering point, Executive Order No. 56 was signed in 2018 (28). However, different facilities and services such as hospitals and ambulance services remain independently operated.

Several preventive interventions targeting the behavioral risk factors of Filipinos for NCDs, which include stroke, have been implemented in the Philippines. The smoking cessation program of the DOH is one such intervention that includes giving advice to patients at the primary care level and referring to quit clinics at higher levels of care. In addition, the protocol of DOH provides for possible pharmacologic, psychological, and behavioral interventions to support the patient to stop smoking (29). In terms of physical activity, DOH made efforts in its promotion. However, there is a general lack of facilities or places for physical activity due to urbanization. Several efforts were initiated to address unhealthy diets, such as mass media campaigns and behavioral change communication and counseling (12). Furthermore, the Philippine Package for Essential Non-communicable Diseases (PhilPEN) provides lifestyle advice, particularly counseling on diet, physical activity, and smoking cessation, to patients at risk of developing NCDs. The package also provides for managing hypertension, focusing on lifestyle advice and the provision of drugs to control blood pressure (30).

## Health Workforce

Human resources for health essential to the stroke care system include neurologists, neurosurgeons, physiatrists, stroke nurses, and other health professionals. In 2019, the Philippine Neurological Association reported over 400 adult board-certified neurologists (31) and over 100 physiatrists. According to Navarro et al. in a country of 109 million Filipinos, there is only one neurologist catering to the 330,000 population (4). Despite having a low number of professionals, there is an existing disparity in the distribution of specialists across the country as many of them are private practitioners, and 80% of the country's neurologists are concentrated in the Luzon mainland leaving only 12 and 8% of these specialists practicing in Visayas and Mindanao, respectively (24, 31).

The health workforce for stroke care is an essential driver for the thrombolysis of patients with acute ischemic stroke. With 53 acute stroke ready hospitals and about 400 neurologists, the national thrombolysis rate ranges only between 2.40% in government and 3.33% in private hospitals (23, 32).

To augment the need for human resources in stroke care, the Stroke Society of the Philippines together with the World Stroke Organization have conducted a five year nationwide stroke training to aid in organizing stroke teams and developing acute stroke ready hospitals and acute stroke units. Both doctors and nurses from different hospitals across the country joined the nationwide rollout of the training (33). Apart from this, stroke topics were already incorporated in medical curricula, including opportunities for exposure in attending to stroke patients in the emergency rooms and wards. In addition, several neurology residency programs and stroke fellowship programs are available in the country through neurology training institutions (34).

## Health Information Systems

At the moment, stroke is included among the domains of the Philippine Department of Health – Unified Disease Registry System. However, information shared by the different health facilities, mainly from the government, remains limited. This limitation prompts the different hospitals to transmit data in different capacities resulting in a fragmented reporting system. Consequently, the said system generates less accurate estimates of the incidence and prevalence of stroke cases in the country.

## Discussion

There is high mortality and morbidity of stroke in the Philippines, especially in areas outside the major cities where access to quality stroke care is limited. The geographical barrier to access further aggravates the gaps in the building blocks of the healthcare system. Addressing these gaps through a systems approach cutting across governance, financing, and service delivery, among others, is a critical precursor to improving overall stroke outcomes in the country.

The passage of the national policy framework for stroke prevention, treatment, and management paves the way to consolidate and strengthen efforts for improving stroke care in the country. This should be integrated as top priority of the health sector (10). While the policy contains provisions on streamlining protocols and care pathways for stroke, improving hospital capacity, health communication and promotion, and strengthening a national stroke registry, these must be translated into concrete actions to improve stroke outcomes. The China Stroke Prevention Project Committee (CSPPC) actively promoted stroke prevention and control by stroke screening and follow-up, establishment of stroke center network and 1 h gold rescue circles to decrease stroke time lines (35). A similar program for the Philippines may greatly improve stroke care. However, a consistent government financial support coupled with organized system of care is needed for this change.

Alongside the policy, the government must continue to invest in improved stroke care by optimizing financing health services and packages through its national health insurance. Optimization of financing can include expanding and reviewing existing benefit packages and ensuring that medicines reimbursed are reviewed constantly for their effectiveness and safety. Likewise, financing must include reimbursements for patient-level expenses, but funds should also be allocated to improve system-level components of stroke care such as those mentioned in the national stroke policy (AO 2020-0059) of DOH (10) as well as prevention programs and interventions. Effective fund allocation is possible by integrating plans and initiatives on stroke in the Local Investment Plan for Health (LIPH) or as a performance indicator in the Local Government Unit Scorecard for Health. By full devolution under Executive Order 138 (36), local government units must take part in the implementation of the policy by earmarking appropriate funds for the provision of services and augmenting resources from the national level (i.e., increasing capital outlay for the establishment of stroke care facilities/brain centers and procurement of additional stroke diagnostic equipment) to ensure sustainability.

In addition to policies and fiscal interventions, programs targeting the behavioral risk factors for stroke need to be scaled up to support the continuing decrease in rates of obesity and smoking in the country, as reported in the 2019 Expanded National Nutrition Survey (37). In particular, the local health centers, which are the primary contact of Filipinos for healthcare, must be strengthened to provide appropriate support to the general community, such as lifestyle advice and education on the risk factors. A supportive environment must also be put in place to aid the implemented interventions to prevent the risk factors. For example, the government must invest in green spaces, sporting facilities, parks, sidewalks to ensure adequate access to facilities for conducting physical activities given the lack of a budget allocation for promoting health in urban settings (12). Likewise, developing benefit packages for smoking cessation interventions such as nicotine replacement therapy can further improve access to such services. These steps help augment the current efforts of the country and aid in the continuous decrease of smoking prevalence.

To further support these recommendations, patient awareness must also be improved as it is crucial in ensuring an efficient stroke care system. In general, there is low stroke awareness across several regions in the country. In the SSP Guidelines for the Prevention, Treatment, and Rehabilitation of Stroke, a community survey reported that only 34.4% were knowledgeable on stroke, and respondents even misconstrued the disease as a heart attack (38). Community education through a range of health promotion strategies on primary and secondary stroke prevention are crucial information in ensuring the prevention and management of stroke. In addition, knowledge of the signs and symptoms and potential initial response can be life-saving to patients. These initiatives can decrease the time gap before taking action and result in the timely patient presentation to the available healthcare facilities for treatment or management (39).

Patients' knowledge on when to act for stroke emergencies should be coupled with efficient emergency medical services (EMS). WHO has identified rapid EMS dispatch and rapid EMS system transport, and hospital pre-notification as key considerations in maximizing stroke patient recovery. In a study by Millin et al. effective rapid dispatch and access to EMS and stroke care are possible through the leadership of medical directors. Furthermore, such strategies include the assignment of catchment areas and criteria for identifying patients that need transportation. Defined pathways should also be available if a facility cannot accommodate the patient and secondary transfers are necessary (40).

As the Philippine health system transitions to universal health care (UHC), establishing a stroke referral process and plan should be integrated into the healthcare provider network. Identification and coordination of referral centers with an organized stroke team that adheres to standard stroke care recommendations of the World Stroke Organization can contribute to achieving an efficient treatment pathway (41).

On the other hand, in communities where neurologists are inaccessible, non-neurologists can augment the gap in service delivery through the facilitation of telestroke. Telestroke can improve patient outcomes through access to stroke specialists and increasing thrombolysis rate and has been shown to have comparable outcomes compared with a non-telestroke group of patients (42). Investment in telestroke can yield optimal results, such as the case of Finland, where telestroke has resulted in a two- to three-fold increase in thrombolysis and producing comparable outcomes vs. an experienced stroke center (43). Furthermore, telestroke has been found to be cost-effective when lifelong benefits are considered due to the nature of the disease (44). To complement telestroke, ensuring access even to minimal services such as those of healthcare nurses or lay workers can also make a difference in the prognosis of stroke as stipulated in the health service capacity for stroke checklist of the World Stroke Organization (41). This emphasizes the need for training and capacity-building for stroke among primary healthcare workers at the community level aside from nurses and doctors in the hospital settings.

Responsive to the changing landscape of healthcare in the face of a public health emergency, one way is to conduct regular virtual training of health care workers, especially those in geographically isolated and disadvantaged areas. Training also needs to be complemented with adequate and equitable distribution of health workforce responsive to Filipinos in need of timely treatment and management of stroke, including post-stroke care. With only one neurologist catering to 330,000 Filipinos based on the study of Navarro et al. (4), the Philippines needs to expand its pool of stroke specialists to meet with the global median of the neurological workforce to a population of 3.3–100,000 (45).

Interoperable stroke registry within and among public and private facilities across the country is also vital in improving stroke care. In a pilot stroke registry implemented in Nigeria, there was improved stroke awareness, better CT rate, reduced time to CT, reduced short term mortality, improved training and competence of interns and residents as well as better job satisfaction among neurologists (46). Furthermore, Shahraki et al. reported that a registry system for the acute phase of stroke can be helpful in monitoring the rate of early thrombolytic therapy (47). Hopefully, by establishing a national stroke registry through the national stroke policy, better data repository on stroke can provide more insightful data that can be utilized to assess, monitor, and evaluate the effectiveness of available and new interventions and responsiveness of referral pathways in different localities. Furthermore, a more robust data infrastructure can facilitate better estimation of the prevalence and characterization of the risk factors of the disease.

With these recommendations, the systems approach, which includes active monitoring of indicators of the national stroke policy, can significantly improve stroke outcomes in the Philippines.

## Author Contributions

MC, YZ, and DU took the lead in preparing the draft manuscript for publication. All authors participated in the data collection and analysis as well as provided input in developing the manuscript and approved the final version submitted.

## Conflict of Interest

The authors declare that the research was conducted in the absence of any commercial or financial relationships that could be construed as a potential conflict of interest.

## Publisher's Note

All claims expressed in this article are solely those of the authors and do not necessarily represent those of their affiliated organizations, or those of the publisher, the editors and the reviewers. Any product that may be evaluated in this article, or claim that may be made by its manufacturer, is not guaranteed or endorsed by the publisher.

## Abbreviations

CT: computed tomography
DOH: Department of Health
MRI: magnetic resonance imagine
PhilHealth: Philippine Health Insurance Corporation
WHO: World Health Organization.
